# A Critical Role for MAPK Signalling Pathways in the Transcriptional Regulation of Toll Like Receptors

**DOI:** 10.1371/journal.pone.0051243

**Published:** 2013-02-06

**Authors:** Marylene Y. Peroval, Amy C. Boyd, John R. Young, Adrian L. Smith

**Affiliations:** 1 Institute for Animal Health, Compton, Berkshire, United Kingdom; 2 The Jenner Institute, Old Road Campus Research Building, University of Oxford, Nuffield Department of Clinical Medicine, Oxford, Oxfordshire, United Kingdom; 3 Department of Zoology, University of Oxford, Tinbergen Building, Oxford, Oxfordshire, United Kingdom; University of Cambridge, United Kingdom

## Abstract

Toll-like Receptors (TLR) are phylogenetically conserved transmembrane proteins responsible for detection of pathogens and activation of immune responses in diverse animal species. The stimulation of TLR by pathogen-derived molecules leads to the production of pro-inflammatory mediators including cytokines and nitric oxide. Although TLR-induced events are critical for immune induction, uncontrolled inflammation can be life threatening and regulation is a critical feature of TLR biology. We used an avian macrophage cell line (HD11) to determine the relationship between TLR agonist-induced activation of inflammatory responses and the transcriptional regulation of TLR. Exposure of macrophages to specific TLR agonists induced upregulation of cytokine and nitric oxide pathways that were inhibited by blocking various components of the TLR signalling pathways. TLR activation also led to changes in the levels of mRNA encoding the TLR responsible for recognising the inducing agonist (cognate regulation) and cross-regulation of other TLR (non-cognate regulation). Interestingly, in most cases, regulation of TLR mRNA was independent of NFκB activity but dependent on one or more of the MAPK pathway components. Moreover, the relative importance of ERK, JNK and p38 was dependent upon both the stimulating agonist and the target TLR. These results provide a framework for understanding the complex pathways involved in transcriptional regulation of TLR, immune induction and inflammation. Manipulation of these pathways during vaccination or management of acute inflammatory disease may lead to improved clinical outcome or enhanced vaccine efficacy.

## Introduction

Toll-like receptors (TLR) constitute the best-characterised family of pattern recognition receptors (PRRs) of the innate immune system. TLRs recognize conserved microbial motifs, known as microbe-associated molecular patterns (MAMPs) broadly expressed by bacteria, fungi, protozoa and viruses. TLR activation is critical for induction of innate and adaptive immune responses. However, since inappropriate activation of TLR can be harmful, the response is tightly regulated. To date, 10 members of the TLR family have been identified in humans, 13 in mice and 10 in chickens. Many aspects of TLR biology are conserved between these vertebrate species, including the repertoire of TLRs, their agonist specificities, the signalling pathways engaged, and the consequences of activation [1], [2], [3], [4]. In birds, TLR3, 4, 5 and 7 are orthologous to mammalian TLR. The TLR1/2 family are also conserved although there are two TLR1-like and two TLR2-like genes in birds which share agonist specificities with the mammalian TLR1/2 family [5], [6], [7], [8]. The avian TLR8 gene is disrupted, but avian TLR7 recognises all known agonists of mammalian TLR7 and 8. The inflammatory response of chickens to oligodeoxynucleotides containing CpG motifs (CpG ODN) is well documented [9], [10], [11]. CpG ODN is recognised by the avian TLR21 molecule, compensating for the lack of a TLR9 orthologue in the avian genome [12]. Avian TLR15 is not found in mammals and is phylogenetically distinct from other vertebrate TLR [2].

TLR activation initiates complex signalling cascades which result in a range of pro-inflammatory events, depending on the TLR and cell type involved [13]. Signalling through the MyD88 dependent and independent pathways is largely conserved between mammals and chickens, although chicken TLR4 may not signal through the MyD88 independent pathway [14]. In macrophages, evolutionarily conserved signal transduction pathways have been shown to mediate inflammatory processes including mitogen-activated protein kinase (MAPK) induced effector mechanisms [6], [15], [16] There are three major groups of MAPK: the extracellular signal-regulated protein kinase 1/2 (ERK), the p38 MAP kinases (p38), and the c-Jun amino-terminal kinase (JNK), which differentially regulate many cellular functions including inflammation [17]. Several TLR induce the Phosphoinositide-3 kinase (PI3K) – Akt pathway which can regulate the immune response in a negative or positive manner [18]. The glycogen synthase kinase 3 (GSK3) is a downstream target of PI3K-Akt signalling and is responsible for regulating cytokine production after TLR activation [19], [20]. Other signalling molecules implicated in TLR-mediated responses included the Janus kinase (JAK) family, the double-stranded RNA (dsRNA)-activated serine/threonine kinase R (PKR) protein and the serine/threonine kinase Protein kinase A (PKA) [21], [22], [23], [24]. Hence, the outcome of TLR activation is a consequence of interactions between multiple signalling pathways, many of which interact to fine-tune the inflammatory response [25].

Most studies of the modulation of TLR levels following activation with agonists have focussed on the agonist that interacts with the TLR under study, such as the modulation of TLR4 after exposure to bacterial lipopolysaccharide (LPS) [26]. For clarity, we refer to these events as cognate interactions. Over-activation of TLR4 with LPS leads to acute systemic disease known as endotoxic shock [27]. Endotoxin tolerance is a well described phenomenon involving TLR4 modulation, whereby cells exposed to LPS become less responsive to a continued or repeated exposure to this agonist [28], [29]. This may help to avoid the pathology associated with uncontrolled inflammation. Similar tolerogenic effects have been reported with a wide range of agonist-TLR combinations [30], [31], [32]. Agonist tolerance can be mediated by a variety of mechanisms, including induction of negative regulators of TLR signalling pathways, such as SIGRR or Tollip [33], [34] and by modification of the availability of TLR molecules and/or other signalling components [35]. Agonist tolerance-associated changes in TLR availability may occur as a result of transcriptional or post-transcriptional effects. During the last few years, some reports have also documented non-cognate regulation of TLR responsiveness (also known as cross-tolerance), including the effects of R848 exposure on the levels of TLR9 [36] or CpG ODN effects on TLR2 and TLR4 expression [37]. Other reported inter-TLR interactions include those between TLR2 and TLR4 [38], and betweenTLR2 and TLR5 [39]. Agonist exposure can lead to a reduction or increase in TLR levels, depending on many factors, including species, cell type and TLR in question [40], [41]. Few studies have identified which signalling pathways contribute to the modulation of TLR expression. In mice, stimulation of TLR1/2 with PAM3CSK4 leads to JNK activation which triggers a positive feedback cascade, increasing the expression of TLR1 [42], [43]. In contrast, inhibition of the ERK pathway during TLR4 stimulation provoked the up-regulation of TLR2 expression, suggesting that ERK activation has a negative effect on TLR2 expression in macrophages [43]. The availability of inhibitors that act on specific components of the TLR signalling cascade has been instrumental in determining which of those components are involved in production of inflammatory mediators and can be applied to identify the pathways associated with TLR regulation.

No comprehensive analysis of TLR regulation (both cognate and cross-regulation) in a single cell type has been undertaken hence we explored this question using chicken macrophages. This system was chosen as a representative vertebrate species (which can be compared with others in the future) that has intrinsic relevance to global food security. We established the existence of a complex network of cognate and cross-TLR transcript regulation and used pharmacological inhibitors of selected signalling components to determine the major pathways involved in this process.

## Results

The macrophage cell line, HD11, [44] expressed a broad spectrum of TLRs (all tested) and responded to a range of agonists in a similar manner to *ex-vivo* monocyte derived macrophages (Figure S1). An investigation of the dynamics of agonist-induced responses demonstrated that exposure to PAM, FSL, LPS, flagellin, R848 or CpG ODN induced up-regulation of IL1β, IL-6 and iNOS, mRNA and NO from 10 hours post-exposure (Figure S2A-B). TLR3 mediated agonist responses, detected by IFNβ mRNA induction, were transient, detectable only at 1 h and 2 h post-stimulation (Figure S2C).

### Signalling pathways involved in TLR mediated pro-inflammatory response

**Figure 1:**
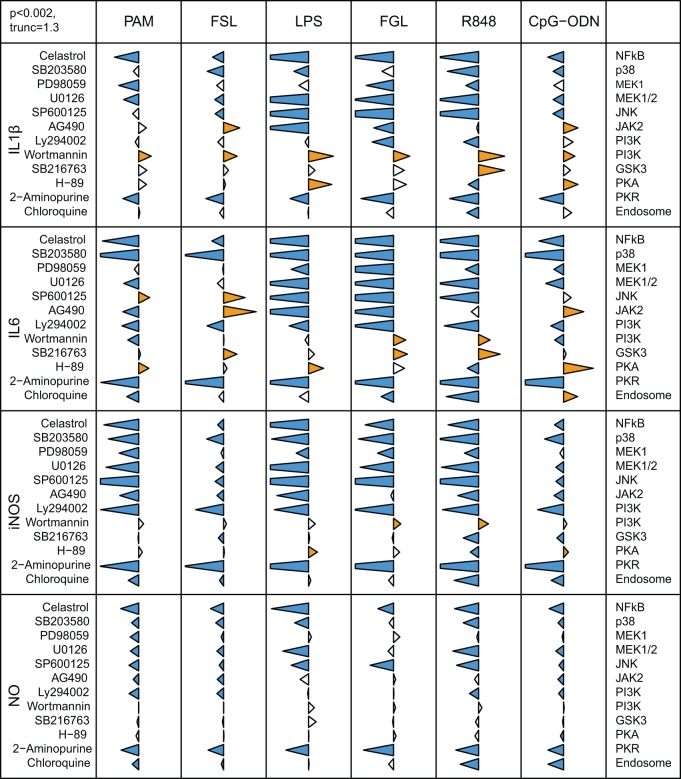
Differential involvement of signalling pathways in TLR mediated responses. HD11 cells were pre-treated with inhibitors and then incubated with TLR agonists. Expression of IL1β, IL6 and iNOS was assessed by qRT-PCR. NO concentrations in cell supernatants were determined using the Griess reagent system. The data for each inflammatory mediator are represented in relation to the agonist employed (top) and the inhibitor (left); with the target indicated on the right. Tips of triangles represent the ratio of agonist response in the presence of inhibitor to that in its absence, on a logarithmic scale. The triangles are truncated on the left at −1.3 units (95% inhibition) and on the right at 1.3 (20 fold enhancement). Blue and orange triangles indicate significant inhibition or enhancement, respectively (p<0. 002, two sided t-test with Holm correction for multiple testing).

Agonist driven TLR stimulation consistently resulted in the up-regulation of IL1β, IL6 and iNOS expression levels as well as in the increase of NO production (Figure S2). The increases were sustained, except in the case of poly(I:C), with which the measurements declined after three hours. In order to identify the signalling pathways involved in the production of these inflammatory mediators, we used well-characterised inhibitors and we assessed changes in cytokine mRNA, iNOS mRNA and NO production (Figure 1).

#### 1) PAM and FSL

The bacterial lipoproteins PAM and FSL are recognised by combinations of TLR1 and TLR2 family members [3], [45], [46]. These agonists induced substantial increases in IL1β and IL6 mRNA levels as well as a significant up-regulation of iNOS mRNA and NO production (Figure S2). The transcription factor NF-κB is recognised as a master regulator of TLR induced responses [47]. NF-κB inhibition led to a reduction in PAM- or FSL- induced pro-inflammatory cytokine mRNA, iNOS mRNA and NO production. Inhibitors of MAPK pathway components p38, ERK (e.g. MEK1 and MEK1/2) and JNK generally impaired induction of expression of IL1β, iNOS mRNA and NO production. ERK or p38 inhibitors reduced agonist-stimulated IL6 mRNA levels while JNK inhibition enhanced induction of IL6 mRNA. Inhibition of the PI3K pathway, a known negative regulator of TLR activity [18], using two inhibitors LY294002 and wortmannin, produced contrasting data. LY294002 pre-treatment led to decreased expression of pro-inflammatory cytokines and nitric oxide. Wortmannin did not affect the nitric oxide pathway, but enhanced levels of mRNA encoding IL1β. Wortmannin did not affect FSL induced IL6 mRNA levels but impaired PAM-induced IL6 induction. JAK2 is recognised as an upstream activator for both JNK and PI3K signalling pathways [22], [48]. Inhibition of JAK2 resulted in increased IL1β and IL6 mRNA induction following FSL stimulation, but led to decreased iNOS mRNA levels and NO production. In contrast, JAK2 inhibition led to a reduction of all PAM-induced responses with the exception of IL1β. GSK-3 is a downstream target of the PI3K-Akt signalling pathway and has been reported to be involved with regulation of pro-inflammatory and anti-inflammatory cytokines [20]. Inhibition of GSK3 activity led to a slight increase in PAM and FSL induction of IL1β and IL6 mRNA. However, the impairment of GSK3 signalling did not affect the nitric oxide response induced by PAM and provoked only a slight decrease in iNOS levels following FSL stimulation. Inhibition of PKA had no significant effect on cytokine expression induced by PAM or FSL, except for up-regulation of IL6 during PAM stimulation. The PKR protein is mostly known for its role in dsRNA induced TLR activation, although PKR activation is also linked with responses to extracellular stress, cytokines or bacterial products [49]. Inhibition of PKR substantially reduced expression of IL1β, IL6 and iNOS mRNAs and NO production upon stimulation with both PAM and FSL. Although TLR1 or TLR2 are not known to be located in the endosomes, chloroquine treatment had a small but significant negative impact on PAM induced IL6 and NO responses.

#### 2) LPS

TLR4 recognition of LPS induced a strong cytokine and NO response (Figure S2). As expected, inhibition of NF-κB led to a dramatic reduction of these LPS-induced responses. Application of specific inhibitors for p38, ERK, JNK or JAK2 abrogated LPS induction of IL1β, IL6 and iNOS mRNA expression and reduced LPS induced NO production. Wortmannin mediated inhibition of PI3K activity notably enhanced LPS induced IL1β expression and but did not effect significant changes in IL6 mRNA or NO responses. In contrast, LY294002 mediated inhibition of PI3K activity reduced LPS induction of IL6 and iNOS mRNAs. Inhibition of PKA activity led to increased IL1β, IL6 and iNOS mRNA induction by LPS, whereas blockade of PKR activity abrogated expression of the inflammatory cytokines, iNOS and NO. Application of GSK3 inhibitor or chloroquine did not significantly affect the observed responses.

#### 3) FGL

Application of the TLR5 agonist FGL promoted increases in the levels of pro-inflammatory mediator mRNA and NO (Figure S2). Inhibition of NF-κB signalling abrogated all responses. The decrease of IL1β, IL6 and iNOS mRNA achieved by impairment of p38, MEK1/2 and JNK indicated the importance of MAPK signalling pathways during FGL mediated response. Inhibition of JAK2 activity induced a significant down-regulation of IL1β and IL6 mRNA induction but did not affect the NO pathway. As observed with other agonists, inhibition of PI3K activity by treatment with LY294002 and wortmannin had different effects on FGL-TLR5 induced responses. Treatment with LY294002 substantially reduced IL1β, IL6 and iNOS mRNA induction, whereas treatment with wortmannin led to a significant increase. SB216763 (GSK3 pathway) induced a significant up-regulation of IL6 mRNA induction and a slight up-regulation of IL1β. Suppression of PKA activity induced a consistent up-regulation of IL1β and IL6 mRNA induction although this was not statistically significant. Inhibition of PKR activity induced strong and significant reduction of IL1β, IL6, iNOS mRNA and NO induction. Unexpectedly, chloroquine treatment moderately impaired FGL-induced up-regulation of IL6 mRNA levels.

#### 4) R848

TLR7 mediates responses to single stranded RNA and various synthetic nucleotide analogues such as R848 [50], [51]. Inhibition of NF-κB activity abolished expression of IL1β, IL6, iNOS mRNA and NO induction by R848. Likewise, inhibition of p38, MEK1, MEK1/2 and JNK impaired IL1β, IL6 and iNOS expression, as well as NO production, induced by R848. This suggests an important role for the MAPK pathway in the TLR7 mediated response. Simultaneous inhibition of MEK1 and MEK2 resulted in a more potent inhibitory effect that the use of PD98059 alone. AG490 affected induction of iNOS, but not the other R848 responses.

Inhibition of PI3K with LY294002 reduced the levels of cytokine and iNOS mRNA and NO production in response to R848. In contrast, wortmannin-mediated inhibition of PI3K activity led to up-regulation of IL1β, IL6 and iNOS mRNA induction. Inhibition of GSK3 also enhanced R848 induction of IL1β and IL6 mRNAs, but reduced induction of iNOS mRNA. Inhibition of PKA and PKR activity or endosomal acidification during R848 responses led to a strong reduction of IL1β, IL6, iNOS induction and, with the exception of PKA, to significant decrease in NO induction.

#### 5) CpG-ODN

Inhibition of NF-κB, MAPK and PKR signalling pathways impaired the inflammatory cytokine and NO pathways induced by CpG-ODN. The JAK2 inhibitor AG490 impaired induction of iNOS mRNA and NO, but led to significantly increased induction of IL1β and IL6 mRNA. The PKA inhibitor H-89 enhanced induction of IL1β, IL6 and iNOS mRNA. The PI3K inhibitor wortmannin increased up-regulation of IL1β expression by CpG-ODN. The alternative PI3K inhibitor Ly294002 reduced induction of IL6 mRNA by this agonist, and also impaired NO pathways.

#### 6) Poly(I:C)

Poly(I:C) is a synthetic mimic of viral dsRNA and induces immune responses similar to those seen during viral infection [52] through the activation of TLR3. Stimulation with Poly(I:C) induced significant expression of IFNα and IFNβ (Figure S2C). Inhibition of NF-κB reduced the induction of IFNβ mRNA, as did treatment with AG490, Ly2940002 or 2-aminopurine. IFNα induction was significantly reduced by AG490 or 2-Aminopurine.

### Signalling pathways involved in modulation of TLR expression upon stimulation with cognate agonist

**Figure 2:**
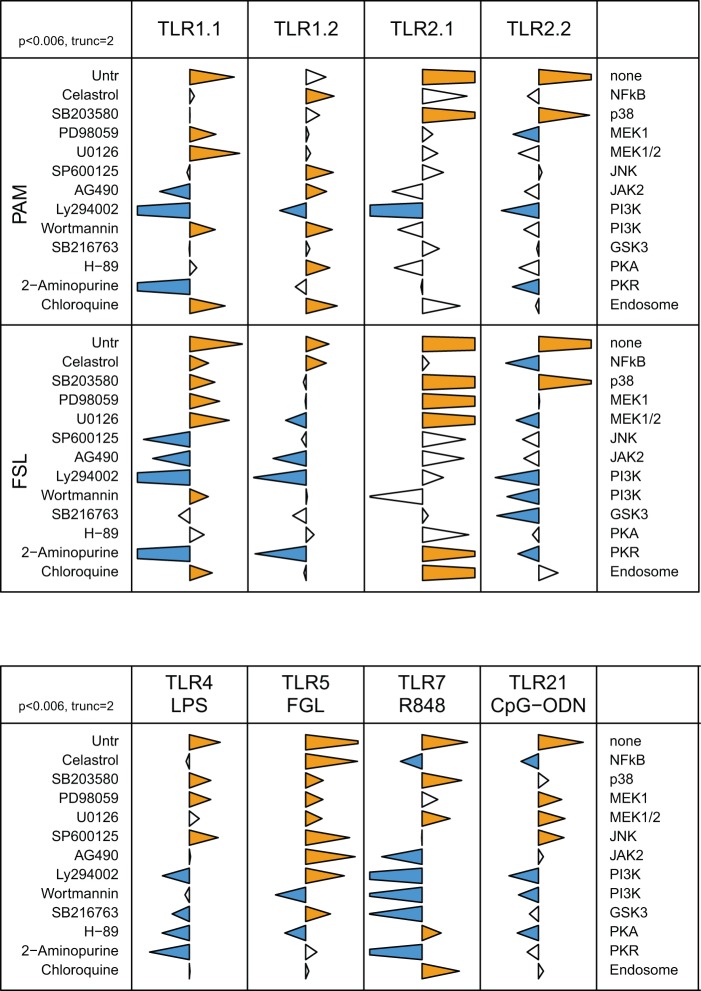
Differential involvement of signalling pathways in cognate regulation of TLR expression. HD11 cells were pre-treated with inhibitors and then incubated with TLR agonists or media (Untr). Expression of cognate TLR mRNA was assessed by qRT-PCR. The data for each inflammatory mediator are represented in relation to the agonist employed (top) and the inhibitor (left); with the target indicated on the right. Tips of triangles represent the ratio of TLR expression in the presence of inhibitor to that in its absence, on a logarithmic scale. The triangles are truncated at 2 units either side of the axis (99% inhibition on the left and 100 fold enhancement on the right). Blue and orange triangles indicate significant inhibition or enhancement, respectively (p<0.006, two sided t-test with Holm correction for multiple testing).

**Figure 3:**
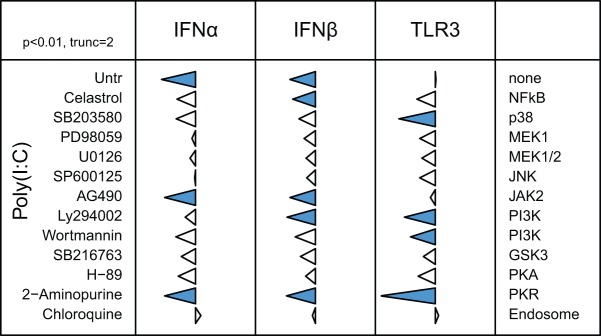
Signalling pathways involved in TLR3 mediated responses. HD11 cells were pre-treated with inhibitors and then incubated with Poly(I:C) or medium (Untr). Expression of IFNα, IFNβ and TLR3 was assessed by qRT-PCR. Measured levels, shown by the tips of the triangles, are relative to those in cells treated with poly(I:C) but without inhibitor. The triangles are truncated on the left at 2 units. Blue triangles indicate significant differences from the induced but uninhibited level (p<0.01, two sided t-test with Holm correction for multiple testing).

Stimulation of HD11 cells with all agonists except of poly(I:C), induced down-regulation of the cognate TLR mRNA. Using the inhibitor-based approach, we elucidated the signalling pathways involved in these events. Results are displayed in figures 2 and 3.

#### 1) PAM and FSL

PAM and FSL down-regulated expression of the mRNA encoding members of the TLR1/2 family. Inhibition of MEK1/2 signalling partially or wholly rescued TLR1.1 down-regulation induced by both lipoproteins, while inhibition of p38 signalling only altered TLR1.1 down-regulation by FSL but not that by PAM. The partial rescue of TLR1.1 expression by wortmannin and chloroquine was the same for both agonists. In contrast, the down-regulation of TLR1.2 expression was not affected by the MEK pathway inhibitors for either agonist. TLR1.2 down-regulation was rescued by SP600125, AG490, wortmannin, H-89 and chloroquine during treatment with PAM, but not with FSL revealing agonist-specific aspects of the regulation of this TLR.

Regulation of TLR2.1 expression was similarly agonist-specific. Only inhibition of p38 rescued TLR2.1 down-regulation after PAM treatment, while TLR2.1 down-regulation by FSL was rescued by inhibition not only of p38, but also of MEK1/2, PKR and endosomal acidification. In contrast to the agonist-specific regulation of TLR2.1, rescue of TLR2.2 expression was only by inhibition of the p38 pathway during either PAM or FSL treatment. Chloroquine partially rescued TLR2.2 down-regulation in the presence of FSL.

#### 2) LPS

Cognate down-regulation of TLR4 mRNA expression by LPS was reversed by inhibition of p38, MEK1 and JNK signalling pathways. Inhibitors of other signalling pathways, some of which are essential for cytokine or NO production, did not recue TLR4 mRNA levels, some even decreasing it further.

#### 3) FGL

Reduction in TLR5 mRNA levels by exposure to FGL was highly dependent on NF-κB activity. MAPK inhibitors partially restored this down-regulation, although interference with JNK activity was more effective than interference with p38 or MEK1/2. In addition, inhibition of JAK2 activity rescued TLR5 mRNA down-regulation. Ly294002 and SB216763 (PI3K and GSK3 inhibitors) rescued some down-regulation of TLR5 mRNA, whereas wortmannin (PI3K inhibitor) did not.

#### 4) Nucleic acid-recognising TLR

TLR3 mRNA levels were not affected following stimulation with cognate agonist (Figure 3). Blockade of p38, MEK1/2 and PKA pathways rescued TLR7 mRNA levels. As expected, inhibition of endosomal acidification rescued the R848-induced down-regulation of TLR7 mRNA levels. TLR21 mRNA levels were rescued by inhibition of the MEK and JNK signalling pathways (Figure 2).

### Cross regulation of TLR expression during PAMP stimulation

**Figure 4:**
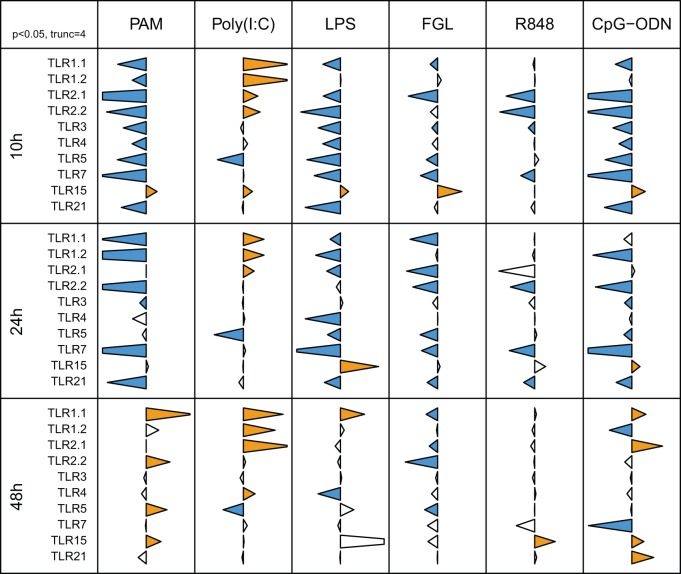
Agonist induced cross-regulation of TLR mRNA levels. HD11 cells were stimulated with agonists for 10, 24 and 48 h and the levels of all TLR mRNA were measured in all samples. Data are represented in relation to the agonist employed (top) and the analysed TLR (left) at different times. The tips of triangles represent the change in adjusted Ct for the indicated TLR mRNA (inner rows) after exposure of HD11 cells to the indicated agonists (columns) for the indicated times (outer rows), reduced expression to the left and increased expression to the right using a logarithmic (Ct) scale. Triangles are truncated at +/−4 Ct units (about 16-fold difference). Triangles coloured orange or blue indicate significant increase or decrease in expression (Welsh's two-sided t test; p<0.05 after correction for multiple testing using “fdr” method of Benjamini and Hochberg [99]).

Systematic analysis with a range of agonists revealed a high degree of both cognate and cross regulation of TLR mRNA expression (Figure 4). All but one of the agonists caused initial down-regulation of the cognate TLR, which was either sustained (TLRs 4, 5, 7) or reversed at 48 hours (1.1, 21). The exception was Poly(I:C) which had no effect on its own receptor. Cross regulation was complex, although the most common effects were either none or initial down-regulation followed by recovery or reversal by 48 hours. A notable exception was poly(I:C), which up-regulated expression of the TLR1/2 family. Poly(I:C) had no effect on other TLRs except for a sustained down-regulation of TLR5. TLRs 3 and 4 were the least affected by non-cognate agonists, both being slightly and transiently down-regulated by PAM and CpG-ODN. The cognate agonist for TLR15 has not been identified. TLR15 was up-regulated by all of the agonists, all of which are classified as non-cognate. A sustained up-regulation of TLR15 mRNA was observed in the presence of LPS.

### The MAPK pathway is involved in cross-modulation of TLR expression

**Figure 5:**
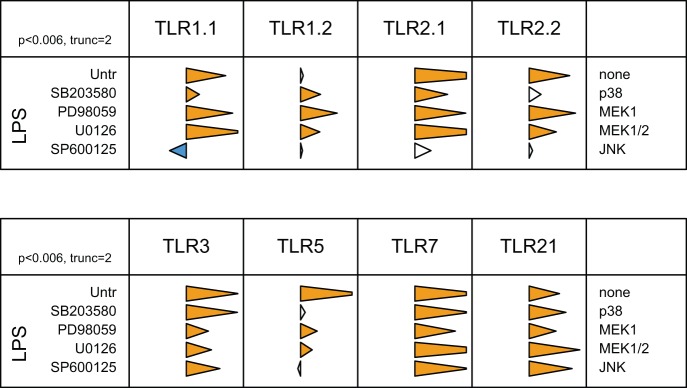
MAPK signalling pathways are responsible for LPS mediated cross-regulation. Pre-treatment of HD11 cells with MAPK inhibitors was followed by LPS stimulation. TLR mRNA levels were assessed by qRT-PCR. The data for each inflammatory mediator are represented in relation to the agonist employed (top) and the inhibitor (left); with the target indicated on the right. Tips of triangles represent the ratio of TLR expression in the presence of inhibitor to that in its absence, on a logarithmic scale with triangles truncated at 2 units either side of the axis. Blue and orange triangles indicate significant inhibition or enhancement, respectively (p<0.006, two sided t-test with Holm correction for multiple testing).

Data demonstrated a consistent role for elements of the MAPK pathway (p38, MEK and JNK) in agonist-dependent regulation of cognate TLR mRNA levels. LPS consistently and significantly affected levels of mRNA encoding several different TLR (Figure 3). Thus, the effect of MAPK inhibition on LPS-induced TLR cross-regulation was assessed (Figure 5). Inhibition of the p38 and MEK signalling pathways reduced the down-regulation of TLR1.1 and both TLR2 induced by LPS treatment. In contrast, JNK was not involved in the rescue of expression of TLR1/2 family mRNA. Although LPS did not down-regulate TLR1.2 mRNA levels, SB203580, PD98059 and U0126 led to the up-regulation of TLR1.2 expression in presence of LPS, suggesting a role for p38 and MEK in modulation of TLR1.2. The LPS-induced reduction in TLR5 mRNA was not affected by exposure to inhibitors targeting JNK or p38, although it was partially rescued by inhibition of MEK activity. LPS stimulation induced the down-regulation of the three TLRs involved in nucleic acid recognition, which was rescued by inhibition of p38, MEK1/2 or JNK.

## Discussion

The spectrum of MAMPs that stimulate macrophages from different vertebrates is highly conserved and is reflected by the high level of conservation of the TLR repertoire [2], [53]. The importance of TLR-mediated induction of immune responses is matched by the need to regulate these responses to avoid pathological reactions. TLR-mediated responses are tightly controlled by mechanisms including regulatory components of signalling pathways, subcellular relocalisation of TLR proteins as well as transcriptional or posttranscriptional modulation [25], [33], [39], [54]. Previous studies have documented regulation of cognate TLR (e.g. TLR4 by LPS to avoid or reduce the impact of endotoxic shock, [28]) and some have extended these to include non-cognate interactions where one TLR agonist affects the biology of a TLR not involved in the recognition of the inducing agonist [37], [39].

Although agonist induced TLR hyporesponsiveness has been documented our studies with HD11 macrophages revealed a level of transcriptional regulation more complex than previously reported. Cognate down-regulation of TLR mRNA levels was detected after exposure of HD11 macrophages to LPS (with TLR4), PAM or FSL (with TLR1/2 family members), flagellin (with TLR5), R848 (with TLR7) and CpG ODN (with TLR21). Previous studies reported changes in TLR4 protein levels on the surface of monocyte-derived macrophages after exposure to LPS although this effect was dependent upon genetic background [55], [56]. With most cognate TLR regulation a reduction in mRNA levels was readily detected at all time points tested (10 to 48 hours post-treatment), although with CpG ODN and PAM the reduction in mRNA levels was more transient (Figure 3). In contrast, poly(I:C) treatment did not affect TLR3 mRNA levels (Figure 3 and [57]). A complex pattern of cross-regulation of TLR mRNA levels was induced by exposure to all of the tested agonists. For example, PAM treatment led to a transient down-regulation of TLR3, 4, 5, 7 and 21 mRNA levels whereas LPS induced transient down-regulation of certain TLR1/2 family members as well as TLR5, 7 and 21. Similarly, complex and distinctive patterns of TLR mRNA regulation were observed with flagellin, R848 and CpG ODN. Remarkably, TLR15, an avian TLR activated by cleavage with microbe-secreted enzymes [58] was regulated differently to most of the other TLR, generally being up-regulated. TLR15 mRNA levels were increased in the caecal tissue from *Salmonella enterica* serovar Typhimurium (STM)-infected chickens or in polymorphonuclear cells and fibroblasts exposed to STM [59], [60]. Further indication of the complexity of TLR mRNA regulation was that poly(I:C) signalling via TLR3 induced up-regulation of TLR1/2 family members whereas these were down-regulated or unaffected by other agonists. In contrast, a range of other agonists, including poly(I:C), induced down-regulation of TLR5 mRNA levels. Despite the lack of cognate modulation for TLR3 mRNA levels, other TLR agonists induced transient reduction of TLR3 mRNA levels. Collectively, these data indicate a complex inter-relationship between TLR agonist exposure and regulation of TLR mRNA levels, with effects on cognate TLR mRNA as well as mRNA encoding other TLR. These changes were evident between and within families of TLR with diverse agonist recognition profiles. Cognate and cross-regulation of TLR mRNA have also been reported in mammalian cells, although the patterns may differ between species and/or cell type [39], [61], [62], [63], [64]. For example, in murine macrophages, LPS induces an up-regulation of TLR2 and CpG-ODN up-regulates TLR4 [64] whereas in our study with HD11 cells, exposure to LPS or CpG-ODN induced down-regulation or TLR2 and TLR4. Although species and cell type specific differences will be important in particular contexts, the extensive cross-regulation of TLR is an evolutionarily conserved phenomenon.

Regulation of TLR agonist mediated responsiveness is widely documented and may be influenced by cytokines [65], [66], [67], soluble decoy TLR [68], [69] or by direct involvement of the TLR signalling pathway in a cell autonomous manner [63], [70]. The high degree of conservation in TLR signalling pathways [71] facilitated a pharmacological approach to determining the relationship between induction of pro-inflammatory mediators and TLR regulation. All of the pro-inflammatory mediators were sensitive to the effects of celastrol exposure with all of the TLR agonists, supporting a central role for the transcription factor NF-κB in the inflammatory response [72], [73]. Previous studies with chicken cells also reported either activation of NF-κB by TLR agonists [7], [8], [12], [14], [46] or reduction in NO production after inhibition of NF-κB [74], [75]. Celastrol mediated NF-κB inhibition affected reduction of cognate TLR mRNA with TLR1.1, 1.2 and TLR5 but not with other TLR. In most cases NF-κB was not essential for regulation of TLR mRNA levels suggesting that this process is differentially regulated to the inflammatory response.

The three major MAPK cascade components p38, ERK and JNK play an important role in TLR signalling and are differentially involved in cell maturation and production of pro-inflammatory mediators [76], [77], [78], [79], [80]. Our data confirm the role of ERK in IL6 upregulation after stimulation of heterophils with LPS and FGL [81], [82], but also indicate a role for p38 and JNK. Similarly, inhibitors of MEK1/2, p38 or JNK abolished LPS or FGL induced up-regulation of iNOS mRNA and/or NO production. Inhibition of MEK1/2, p38 or JNK also affected IL-1β mRNA after exposure to LPS or FGL. With other agonists (PAM, FSL, R848 or CpG ODN), inhibition of the MAPK pathways frequently led to decreased IL-1β or iNOS levels and lower NO production. Although inhibition of MEK1/2 or p38 consistently led to a reduction in agonist induced IL6 mRNA levels, inhibition of JNK activity with SP600125 either inhibited (LPS, FGL, R848) or increased (PAM, FSL, CpG ODN) IL6 mRNA levels. With many of the agonists, inhibition of ERK using PD960559 (a MEK1 inhibitor) was less pronounced than with U0126 (a MEK1 and MEK2 inhibitor), suggesting that MEK2 can partially compensate for inhibition of MEK1. These data indicate the importance of MAPK components in TLR agonist-induced induction of pro-inflammatory mediators.

The involvement of MAPK signalling in modulation of TLR expression is poorly defined. Transfection of murine RAW264.7 cells with a dominant negative JNK reduced expression of TLR1 without affecting TLR2 expression. Izadi *et al* demonstrated that TLR1/2 mediated JNK activation initiates a positive feedback cascade that increases the expression of TLR1 [42]. The MAPK signalling pathways are involved in the cognate regulation of expression of all TLR in HD11 cells. Inhibition of p38, ERK or JNK rescued the induced down-regulation of expression of TLR4, TLR5 and TLR21 after treatment with LPS, FGL and CpG ODN respectively. However, the relative requirement for ERK, p38 and JNK signalling differed between these TLR. With TLR4, all three MAPK components were equally important, with TLR21, JNK and ERK signalling were dominant, whereas with TLR5, JNK was most important. In contrast, R848-induced TLR7 mRNA down regulation was dependent upon p38 and to a lesser extent ERK. With the TLR1/2 family, the MAPK requirements were dependent upon both agonist (PAM or FSL) and the target TLR. After stimulation with PAM, inhibition of ERK completely rescued TLR1.1 mRNA levels. With FSL, ERK was the dominant pathway but inhibition of p38 or JNK partially rescued TLR1.1 mRNA levels. Recovery of TLR1.2 mRNA levels after exposure to PAM was dependent on inhibition of JNK, whereas no MAPK were implicated following exposure to FSL. In contrast, with FSL, p38 and ERK, and to a lesser extent JNK, were involved in rescue of TLR2.1 mRNA levels, while with PAM treatment TLR2.1 rescue was exclusively dependent upon p38. In contrast, rescue of TLR2.2 mRNA levels were entirely dependent upon p38 activity with both agonists, and not affected by inhibition of ERK or JNK.

Inhibition of endosomal acidification by chloroquine treatment has well documented effects upon nucleic acid based agonist recognition via TLR3, 7, 8 and 9 in mammals. Our data indicates that R848 and CpG-ODN mediated activation were chloroquine sensitive, confirming previous studies [10], [12], [50]. The reduction of TLR7 mRNA levels, but not TLR21, was dependent upon endosomal acidification. However, chloroquine also affected cognate regulation of TLR5, TLR1.1, TLR1.2 and TLR2.1 suggesting a more substantial role for chloroquine-sensitive endosomal signalling with non-nucleic acid agonist-TLR combinations than previously considered.

Although double-stranded RNA (dsRNA)-activated serine/threonine kinase R (PKR) is activated by viral-derived cytoplasmic dsRNA, stress or cytokines [49], several reports indicate PKR involvement in the response to bacterial MAMPs [23], [49], [83]. PKR can mediate activation of NF-κB via the IKK cascade and regulate the activities of p38 and JNK leading to the production of pro-inflammatory proteins [49], [83], [84]. Inhibition of PKR activity with 2-aminopurine reduced or abolished up-regulation of the pro-inflammatory mediators for all agonists tested, and affected cognate regulation of TLR1/2, 4 and 7 but not TLR5 or 21. Our data confirm and extend previous reports implicating PKR in poly(I:C) or CpG ODN dependent IFNβ and NO production, as well as PAM or LPS induced IL6 production [11], [23], [85].

PKA is a serine/threonine kinase consisting of subunits with different biochemical properties. Activation of PKA by increasing the intracellular level of cAMP represents one mechanism for modulating immune receptor signalling. PKA can interact with the NF-κB and MAPK pathways at different levels [86], [87] although the involvement of PKA in TLR activation is poorly documented [88]. Inhibition of PKA activity with H-89 had a wide range of effects on agonist induced production of pro-inflammatory mediators and mild effects on the modulation of some cognate TLR regulation (Figures 1 and 2). With most agonists, H-89 treatment increased up-regulation of IL1β, IL6 and iNOS expression, indicating a regulatory role for PKA during TLR activation. In contrast, with R848 induced TLR7 activation, inhibition of PKA reduced expression IL1β, IL6 and iNOS. Previous reports have demonstrated differential effects of PKA with suppression of LPS-induced production of TNF-ά and enhanced production of IL10 [24], [89].

Activation of members PI3K family leads to transient accumulation of phospholipids in cell membranes and activation of pathways responsible for controlling many cellular events, including proliferation, differentiation and survival. Activation of PI3K provokes relocation and phosphorylation of protein kinase B (also called Akt) which can de-activate GSK3 by phosphorylation [90]. GSK3 differentially regulates the production of pro-inflammatory and anti-inflammatory cytokines by affecting the relative amounts of active transcription factors such as CREB and the p65 subunit of NF-κB leading to a balance between CREB-dependent production of IL-10 and NF-κB-dependent production of pro-inflammatory cytokines [90]. Several papers demonstrate that PI3K has a dual role in TLR signalling, suppressing some pathways and promoting others depending upon which PI3K subunits are involved [19], [91]. Two widely used pharmacological inhibitors of PI3K activity, wortmannin and LY294002 often produce conflicting results that may be a consequence of inhibiting the activity of different sub-units, or may result from non-specific effects of LY294002 [92]. Several papers report that PI3K inhibition mediated by wortmannin enhances TLR-mediated cytokine production whereas LY294002 impairs the response [19], [92], [93], [94]. To explore PI3K-GSK3 signalling wortmannin or LY294002 was used to inhibit PI3K and SB216763 to inhibit GSK3 activity. Wortmannin induced an up-regulation of IL1β, IL6 and iNOS mRNA upon TLR activation, but also rescued agonist induced down-regulation of TLR1 mRNA. Inhibition of GSK3 led to increased levels of IL1β and IL6 mRNA, but had variable or no effect on iNOS expression and NO production. Treatment with LY294002 inhibited expression of cytokine mRNA and NO production. Surprisingly, LY294002 and SB216763 did not alter the cognate regulation of TLR mRNA, with the exception of TLR5. Wortmannin, LY294002 and SB216763 reduced IFNβ mRNA expression in response to poly(I:C). The inhibitors employed did not permit discrimination between the different subunits of the PI3K family which may influence the consequences of TLR activation. Moreover, wortmannin and LY294002 can enhance phosphorylation of p38, ERK and JNK suggesting cross-talk between the PI3K and the MAPK pathways [91], [93], [94], [95].

The Janus kinase (JAK) family are tyrosine kinases with four members JAK1, JAK2, JAK3 and TYK2 which are involved in vertebrate cell growth, survival and development. Classically involved in signalling from cytokine receptors (e.g. IL6R), JAK2 is also implicated in the TLR signalling cascade [96], [97]. Pharmacological studies with AG490 and the use of dominant-negative JAK2 indicated roles in production of inflammatory cytokines, activation of PI3K and in the regulation of JNK phosphorylation [21], [22], [48], [98]. Treatment of agonist stimulated HD11 macrophages with AG490 provoked a variety of effects, in most cases down-regulating cytokine or iNOS mRNA and NO production. However, with FSL and CpG ODN stimulation, AG490 induced increased IL1β and IL6 mRNA levels. The impact of JAK2 inhibition on TLR expression was similar to effects attained by inhibiting selected MAPK or PI3K/GSK3 pathways. Inhibiting JAK2, PI3K or JNK pathways rescued TLR1.2 and TLR2.1 during PAM and FSL stimulation respectively. JAK2 inhibition also provoked rescue of TLR5 mRNA levels, as also seen with LY294002 inhibition of PI3K and GSK3. Taken together these data suggest that JAK2 may interact with both JNK and PI3K signalling pathways.

In summary, agonist induced regulation of cognate TLR mRNA levels was dependent upon different intracellular signalling pathways according to the agonist and the target TLR. The MAPK pathway was critical in both induction of inflammatory mediators and in the control of cognate TLR expression, although the relative importance of ERK, p38 and JNK varied between different TLR. There was a wide diversity in requirements for other elements of the signalling pathways e.g. PKA, PKR, JAK2 and PI3K, although perhaps most notable was that NF-κB was not essential for cognate regulation of most TLR mRNA.

Having determined the relative requirements for different signalling pathways in induction of inflammatory mediators and cognate regulation of TLR mRNA (including the prominent role for MAPK signalling in the regulatory effect) we explored the role of MAPK components in TLR cross-regulation. During cognate TLR regulation different combinations of p38, MEK1/2 and JNK were required for down-regulation of the TLR mRNA. The MAPK pathway was also critical in regulating mRNA levels of non-cognate TLR after exposure of HD11 macrophages to LPS. Interestingly, the relative importance of p38, MEK1/2 and JNK in LPS-induced cross-regulation were different to those involved in cognate TLR4 regulation. Moreover, the MAPK requirements for cross-regulation did not match those determined for cognate regulation of respective TLR. For example, whereas cognate agonist-induced TLR2.2 modulation was rescued by exclusively by inhibition of p38, in the case of TLR4-mediated cross-regulation MEK1/2 was the most important MAPK component. Similarly, cognate TLR5 mRNA down-regulation was almost completely rescued by inhibiting JNK, whereas the rescue of LPS-induced down-regulation of TLR5 was mostly attributable to MEK1/2. Hence, the signalling requirements for cognate- or cross-regulation of TLR expression can be different.

Collectively, our data document an extensive network of interactions that affect TLR mRNA levels and outcomes of TLR activation, with a level of complexity that was previously unappreciated. Notwithstanding additional complexities that may be revealed after exposure to multiple agonists, comparing different cell types, species, strains or interfering with multiple signalling components it is clear that the regulation of TLR mRNA involves subtle changes in a wide variety of signalling pathways. The ability to regulate TLR differentially by cognate and non-cognate TLR agonists as well as the influence of other mediators such as cytokines or interferons [65] allows fine tuning of a critical but potentially dangerous section of the immune system. Interestingly, the regulation of TLR mRNA levels has overlapping but distinct requirements compared with production of pro-inflammatory mediators (e.g. for NF-κB or MAPK elements). These differences suggest that at least some are not dependent on autocrine/paracrine effects and relate to cell autonomous signalling pathways. Detailed elucidation of the signalling pathways responsible for changes in agonist sensitivity in multiple cell types and multiple host species offers an opportunity to understand the integration of TLR signalling and may provide insights into the evolution of complex pattern-recognition signalling networks. From a practical perspective, these data provide a foundation to understand the effects of complex adjuvant formulations, infection-induced inflammation and inflammatory diseases with various aetiologies.

## Materials and Methods

### Cell cultures

The avian macrophage HD11 cell line [44] was cultured in complete RPMI medium (2.4% foetal bovine serum, 2.4% chicken serum and 10% Tryptose phosphate broth) at 37°C. HD11 cells were seeded at 1.5×10^5^ ml^−1^ per well of 96 well tissue culture plates, or 3×10^5^ cells ml^−1^ per well of 24 well tissue culture plate and were used at 70% confluence. For each experiment, three wells were used per treatment and data shown are representative of three separate experiments.

Rhode Island Red chickens were bred and reared at the Institute for Animal Health (IAH, Compton, UK) under conventional conditions. At six weeks post-hatch, blood was collected and monocyte-derived macrophages (MDM) were obtained using a modification of an established procedure [50]. Briefly, heparinised blood was mixed with an equal volume of filtered PBS and the suspension was overlaid onto Histopaque®-1083 (Sigma) at room temperature. After centrifugation at 1200×g for 40 minutes at room temperature, cells at the interface were collected. Following addition of ice-cold PBS, the cells were washed by three successive centrifugations at 1200×g for 4 minutes at 4°C. Cells were then resuspended in RPMI-1640 supplemented with 4% chicken serum, 20 mM Hepes, 10 units ml^−1^ penicillin/streptomycin, 10 units ml^−1^ nystatin and 100 µM L-glutamine. Cell viability was estimated by trypan blue exclusion and 4×10^5^ cells were dispensed into individual wells of 96 well tissue culture plates. After 24 h of culture at 37°C, 4% CO_2_, the adherent cells were washed once in medium and fresh complete medium was added. Five chickens were used for each experiment and cells from each bird were seeded in triplicate wells.

### TLR stimulation

All TLR agonists were obtained from InVivoGen (Bioscience-Autogen, UK) and resuspended according to the manufacturer's recommendations. Cells were co-incubated with the following TLR agonists: Pam3CSK4 (PAM, 1 µg/ml), FSL-1 (FSL, 100 ng/ml), polyinosinic-polycytidylic acid (poly(I:C), 50 µg/ml), *Escherichia coli* LPS (1 µg/ml), *Salmonella thyphimurium* flagellin (FGL, 1 µg/ml), R848 (1 µg/ml), ODN M362 (CpG-ODN, 2 µM). During kinetic experiments over 48 hours, 10 ng/ml of FSL were used.

### Signalling pathway inhibition assay

Inhibitors provided by InVivoGen were used to impair various signalling pathways. Inhibitors were resuspended according to the manufacturer's recommendations and diluted in complete RPMI medium prior use. HD11 cells were pre-treated for 1 h with inhibitors, except for 2-Aminopurine which was applied for 4 h. The following concentrations were used: Chloroquine,100 µM; Celastrol, 4 µM; LY294002, 100 µM; AG490, 100 µM; SP600124, 400 µM; PD98049, 100 µM; U0126, 40 µM; SB203480, 20 µM. After addition of inhibitors, 1 µg/ml of TLR agonist was added and the HD11 cells were incubated for a further 10 h, except for poly(I:C), for which 50 µg/ml was used, and incubation was for 2 h.

### RT-PCR

Subsequent to experimental treatments, RNA extraction and DNA removal were performed using QIAGEN RNeasy Mini Kit and QIAGEN RNase-free DNAse, according to the manufacturer's instructions. RNA concentrations were measured using a NanoDrop ND-1000 spectrophotometer (NanoDrop Technologies). Synthesis of cDNA was performed with the iScript Select cDNA Synthesis Kit (Bio-Rad) according to the manufacturer's instructions. PCR products were generated with 1 µl of heat-inactivated cDNA sample by conventional PCR using a G-storm thermocycler (Gene Technologies, Essex, UK). Real-time quantitative RT-PCR was performed using the Applied Biosystems TaqMan® FAST Universal PCR Master Mix (Applied Biosystems). Amplification and detection of target sequences was performed using the Applied Biosystems 7400 FAST Real-Time PCR System. TLR primers and probes were designed using the Primer Express software program (Applied Biosystems, Warrington, UK). Where possible, primers were designed to span predicted introns in order to distinguish between genomic DNA and cDNA templates. TLR primer and probe sequences are listed in Table S1. Primer and probe sequences for 28S and cytokine assays were as previously published [50]. Primers were obtained from MWG Biotech (Ebersberg, Germany) and the probes labelled with the fluorescent reporter dye 4-carboxyfluorescein (FAM) at the 4′ end and the quencher N,N,N,N'-tetramethyl-6-carboxyrhodamine (TAMRA) at the 3′ end were obtained from Eurogentec (Southampton, UK). Quantification was based on the increased fluorescence detected due to hydrolysis of the target specific probes by the 4′ to 3′ exonuclease activity of the hot-start DNA polymerase during amplification. Normalisation of the reporter signal was achieved through use of the passive reference dye 6-carboxy-c-rhodamine, which is not involved in amplification. Results were expressed as the threshold cycle value (Ct) at which the change in the reporter dye passes a significance threshold (DRn). The threshold level was set to the same value on each plate where a given gene was assayed. Ct values for TLR or cytokine mRNA product for each sample were adjusted using the 28S rRNA Ct value for the same sample. The slopes of a plot of Ct against log10 of the standard dilution series present on each plate where a given gene was assayed were calculated. The slopes of the respective gene of interest (GOI) and 28S dilution series were then used to calculate GOI Ct values and adjust for differences in input total RNA as follows: corrected Ct value  =  Ct + (Nt – Ct') × S/S', where Ct  =  mean of triplicate GOI Ct values, Nt  =  median 28S Ct for all samples within an experiment, Ct'  =  mean of triplicate 28S values of individual sample, S  =  GOI slope and S'  = 28S slope. Results were then expressed as 40-Ct values or in fold change [50].

### Nitrite oxide measurement

The Griess reagent system (Promega) assay was used to indirectly measure NO production in the cell supernatant by quantifying the production of nitrite (NO2−), one of two primary, stable and non-volatile breakdown products of NO. The amount of nitrite present is determined by comparison with a standard curve using solutions of sodium nitrite, following manufacturer's recommendations. The kinetics of NO production were determined using supernatants of 1.5×10^5^ cells.

## Supporting Information

Figure S1 **TLR mRNA expression and TLR activation in monocyte-derived macrophages and HD11 cells.** (A–B) TLR expression in monocyte-derived macrophages (MDM, A) and HD11 cells (B) was analysed by both RT-PCR and qRT-PCR. cDNA was screened for genomic DNA contamination using primers designed across an intron of the chicken avidin gene (f5′GGAAATGGACCAACGATCTG-3′ and r5′-CCCTGGTAGCTTTCCAGTCA-3′). RT-PCR products were analysed by electrophoresis on a 2.0% agarose gel and products were visualized by staining with ethidium bromide (Bio-Rad, Ltd.). Marker lane shows 100 bp DNA ladder (Invitrogen). Although the MDM and HD11 expressed a similar TLR repertoire, TLR5 and TLR2.2 mRNA levels were higher in MDM than in HD11 cells while HD11 expressed more TLR 1.2 mRNA. (C) To assess TLR responses, MDM and HD11 cells were co-incubated for 24 hours with TLR agonists, and IL1β mRNA expression was determined by qRT-PCR. Equal amounts of mRNA from MDM and from HD11 cells were used, and three technical replicates were performed. Bars represent the means ± SD of MDM from ten birds or from two replicate HD11 cultures. Asterisks indicated significant differences between MDM and HD11 cells (2 way Anova with Bonferroni correction for multiple comparisons *p<0.05, **p<0.01*** p<0.001 and **** p<0.0001). MDM and HD11 cells showed a broadly comparable pattern of response to well-known TLR agonists, as judged by induction of IL1β mRNA at 24 h post-stimulation, although the basal and stimulated levels were generally higher in HD11. The HD11 line was less responsive to polyI:C, FGL and R8848 than the ex-vivo macrophages.
(EPS)

Figure S2 **TLR induce a time-dependent response in avian macrophages.** To determine the kinetics of responses to various agonists, TLR agonist-treated HD11 cells were collected at different time and (A) expression of IL1β, IL6 and iNOS mRNAs were assessed by qRT-PCR. (B) In supernatants of agonist-treated HD11 cells, NO concentrations were determined using Griess reagent system at indicated times. (C) IFNβ mRNA expression was estimated by qRT-PCR following stimulation of HD11 cells with different concentrations of poly(I:C) during a kinetics. Dashed line represented value of IFNβ in untreated cells. Data are representative of two experiments and results are shown as means ± SD (2 way Anova with Bonferroni correction for multiple comparisons *p<0.05, **p<0.01*** p<0.001 and **** p<0.0001).
(EPS)

Table S1 **Sequences of primers and probes for qRTPCR.** The table includes oligonucelotide sequences for all primers and probes used in this study and accession number for all target sequences. IL = interleukin, IFN = interferon, iNOS = inducible nitric oxide synthase, TLR = Toll-like Receptor.
(TIF)
