# Supplementary figures and images for: A Critical Role for MAPK Signalling Pathways in the Transcriptional Regulation of Toll Like Receptors
Source: PLoS One. 2013 Feb 6;8(2):e51243. doi: 10.1371/journal.pone.0051243 (PMC3566169; doi:10.1371/journal.pone.0051243)

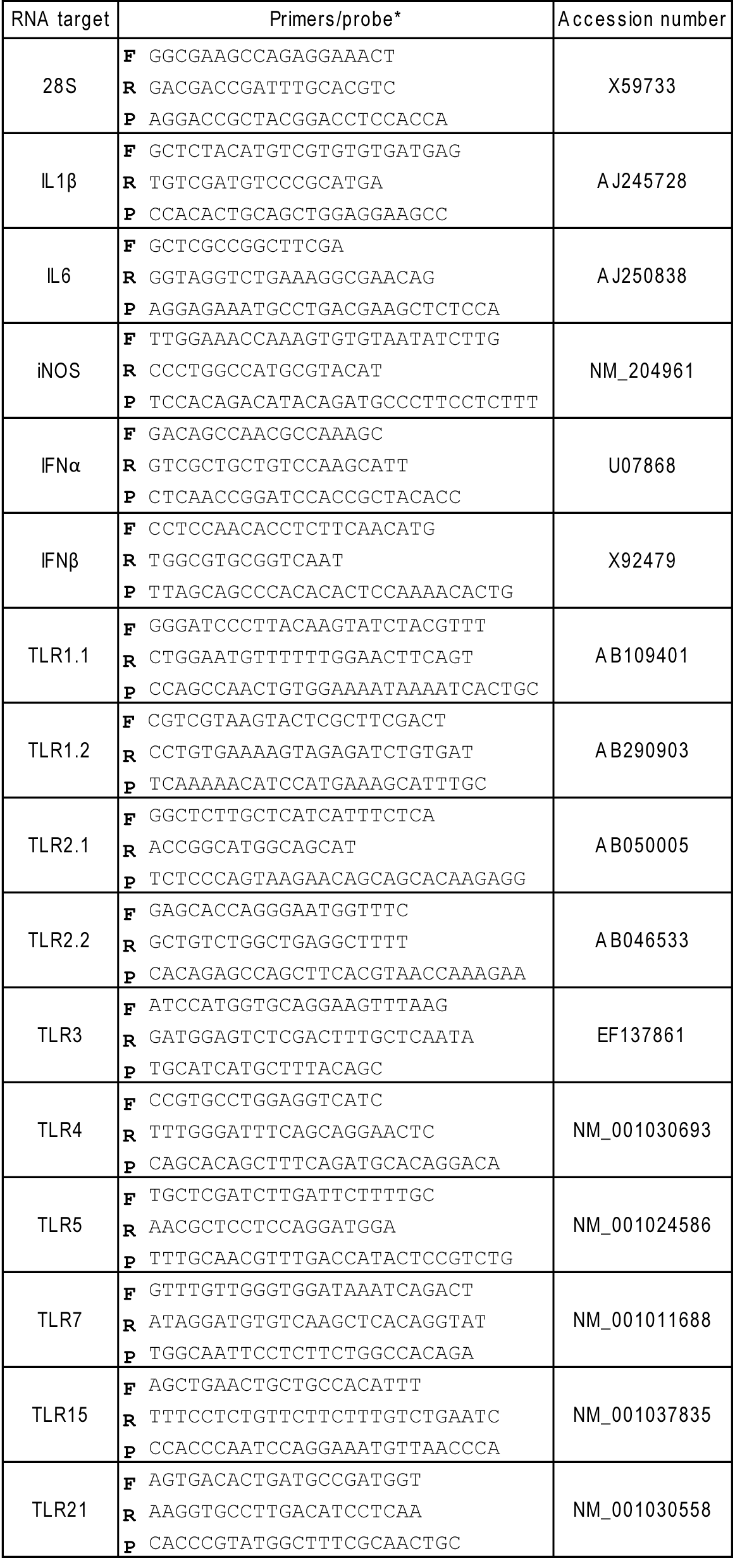

Supplement: Table S1 — Sequences of primers and probes for qRTPCR. The table includes oligonucelotide sequences for all primers and probes used in this study and accession number for all target sequences. IL = interleukin, IFN = interferon, iNOS = inducible nitric oxide synthase, TLR = Toll-like Receptor. (TIF) [file pone.0051243.s003.tif]
